# Measurement and characteristics of the temporal-spatial evolution of China’s healthcare services efficiency

**DOI:** 10.1186/s13690-023-01208-x

**Published:** 2023-11-14

**Authors:** Yizhong Ye, Qunshan Tao

**Affiliations:** 1 School of Hospital Economics and Management, Anhui University of Chinese Medicine, Hefei, 230000 China; 2 Key Laboratory of Data Science and Innovative Development of Chinese Medicine in Anhui Province Philosophy and Social, Hefei, 230000 China

**Keywords:** Healthcare services, Efficiency analysis, Temporal-spatial evolution, Markov chain model, Exploratory spatial data analysis

## Abstract

**Background:**

Healthcare services efficiency (HSE) is directly related to the healthcare demands of the general public and also plays an essential role in the country’s coordinated economic and social development.

**Methods:**

In this study, the stochastic frontier approach (SFA)-Malmquist model was applied to measure the HSE of 31 Chinese provinces based on panel data from 2010–2020. Then, kernel density estimation, Markov chain, and exploratory spatial data analysis were adopted to study the temporal-spatial dynamic evolution characteristics of the HSE.

**Results:**

The study found that China’s HSE showed an average value of approximately 0.841, indicating room for improvement. The HSE varied significantly across regions, presenting an “East > Central > West” distribution layout. The TFP of healthcare services in China grew by 1.6% per year, driven mainly by technological progress of 1.8% per year. The trend of the HSE shifting to a high level in China was significant, but its evolution exhibited stability of maintaining the original state, and it was harder to achieve leapfrog transfer. The temporal-spatial evolution of the HSE was also significantly affected by geospatial factors, with a clear spatial spillover effect and spatial agglomeration characteristics. Provinces with high-level HSE exhibited positive spatial spillover effects, while provinces with low-level HSE had negative spatial spillover effects. Thus, the “club convergence” phenomenon of “high efficiency concentration, low efficiency agglomeration, high levels of radiation, and low levels of suppression” was formed in the spatial distribution.

**Conclusions:**

The results indicate that countermeasures should be taken to improve the HSE in China. Theoretical support for the improvement of HSE is provided in this paper.

**Table:** 

Text box 1. Contributions to the literature
• Literature gaps are still present on how to clearly portray the temporal-spatial evolutionary characteristics of the HSE.
• This national study comprehensively discusses the overall picture of the HSE level and its temporal-spatial evolution in Chinese provinces.
• Regarding temporal evolution, although the overall level of HSE in China has improved, the difference between high- and low-HSE provinces has expanded, and the probability of low-HSE provinces transitioning to high-HSE provinces is considerably low.
• Regarding spatial evolution, China’s HSE exhibits evident spillover effects and agglomeration characteristics.
• These findings contribute to literature gaps that provide theoretical support for governments in improving HSE.

## Introduction

Healthcare services are fundamental to establishing China’s public service system. The level of the healthcare services efficiency (HSE) and the efficiency differences among regions not only restrict the public demand for quality and fair healthcare services to a large extent but also impact the economic development and social stability. The Chinese government has permanently attached importance to the healthy development of the healthcare sector. China’s National Health and Sanitation Commission issued the Action Plan for Further Improvement of Medical Services (2018–2020) in 2017, which specifies the work objectives of promoting the high-quality development of healthcare services and gradually forming a synergistic and integrated regional medical service model [1]. In 2022, the State Council issued the Outline of the 14th Five-Year Plan (2021–2025) for National Economic and Social Development and Vision 2035 of the People’s Republic of China; it asserts that the priority should be enhancing the quality of healthcare delivery and progressively eliminating disparities between urban and rural areas, regions, and people in terms of service capacity and health level [2]. Supported by these policies, China has made significant progress in the field of health care, such as the establishment of healthcare alliances and the improvement of the health insurance system for residents [3, 4]. These changes have given rise to a number of positive impacts, including an increase in health supply capacity and volume levels and a reduction in the burden of access to health care for the population. The number of health technicians per 1,000 people in China increased from 4.37 in 2010 to 7.97 in 2021, and the number of beds in health facilities per 1,000 people has increased from 3.58 to 6.7 during the same period. The proportion of personal health expenditure to total health expenditure in China has also decreased, from 35.3% in 2010 to 27.6% in 2021 [5]. However, there is still a gap between the supply of healthcare services in China and the expanding demand of the masses for high-quality medical services [6], and the medical and health service system still faces problems, including the inadequate and unbalanced allocation of health resources and inefficient medical services [7, 8]. This has severely impeded the healthy growth of China’s healthcare services and has rendered citizens unable to satisfy their medical service demands completely. Moreover, to improve an efficient and high-quality medical and healthcare service system and meet the population’s increasingly diverse medical needs, we must increase the HSE while using limited medical resources and ensuring coordinated development between medical services and the health level in various regions. Thus, effectively improving HSE has become an urgent and essential issue.

Until now, the HSE has been extensively investigated by scholars [9–13]. However, these studies have had shortcomings. First, most studies have typically focused on the micro level (i.e., the efficiency assessment of healthcare institutions such as hospitals, nursing homes, and healthcare service centers), neglecting a macro-level assessment of the efficiency of the entire healthcare delivery system in the country [14]. However, micro-level studies have struggled to clearly reflect the internal heterogeneity of HSE regional development [15]. Second, most scholars have tended to use DEA and its extensions to assess the HSE, but this approach attributes all deviations from the validity boundary to inefficiencies, which affects the accuracy of the analyses [16]. Although the stochastic frontier analysis (SFA) model can overcome these limitations, it has been relatively underutilized in assessing the HSE [17]. Finally, existing studies have mainly concentrated on the measurement, spatial effects, and influencing factors of the HSE, while relevant studies on the features of the temporal-spatial evolution of the HSE have been missing. Further, the study of the temporal-spatial dimensions of HSE will help in the optimization and adjustment of resource allocation and related policies [18]. In summary, after thoroughly considering China's current policy context and existing research gaps, what is the true level of HSE at the macro level in China? What are the characteristics of the temporal evolution of the HSE in China? What is the spatial evolution pattern of the HSE in China? All of these questions deserve further examination.

Based on the above considerations, the objective of this study was to accurately evaluate HSE in all 31 provinces of China at a macro level by utilizing the SFA model and Malmquist indexes and to uncover the regional disparities, temporal-spatial distributions, and evolutionary patterns of the HSE in China by integrating the kernel density estimation, the Markov chain model, and exploratory spatial data analyses. Through this research, we can not only provide a clear outline for policymakers and stakeholders to understand the temporal-spatial features of HSE at the provincial level in China but also optimize the allocation of regional healthcare resources, promote the healthy development of the healthcare industry, and create a practical reference for other countries facing inefficiencies in healthcare services.

Consequently, the main contributions of this study are as follows: (1) It applied the SFA-Malmquist model to assess the HSE of 31 provinces in China from 2010 to 2020 in both static and dynamic dimensions, which makes up for the shortcomings in the research methodology. (2) By combining the kernel density estimation model and the Markov chain model, the time-evolving characteristics such as the distributional differences and transfer trends of HSE in 31 provinces in China were investigated in depth, which expands the related research on HSE. (3) By using exploratory spatial data analysis, we further explored the spatial evolutionary features such as spatial correlation and agglomeration of the HSE in China from 2010 to 2020, which enriches the related research on the HSE.

The remainder of this paper is organized as follows. Section 2 reviews the literature on HSE evaluation and the temporal-spatial features of the HSE. Section 3 describes the methodology, indicator selection, and data sources for this paper. Section 4 provides the findings of the empirical analyses in this study. Section 5 provides the relevant discussion. Section 6 presents the study's conclusions and limitations.

## Literature review

### Efficiency and productivity assessments of HSEs

Efficiency describes the relationship between inputs and maximum outputs per unit of resources, which is an appropriate index for evaluating the level of healthcare delivery [19]. Moreover, accurate and effective efficiency assessments help policymakers and stakeholders formulate and optimize appropriate policies [20]. Thus, the assessment of efficiency and productivity has been the focus of researchers in the healthcare field. However, no consensus exists among scholars on the optimal way to measure efficiency and productivity. In previous studies, nonparametric and parametric approaches are the most popularly employed research methodologies by domestic and foreign scholars. However, the former methods are more frequently used than the latter [20].

Among the nonparametric methods, DEA models and their extended forms are widely used to evaluate the HSE at the macro or micro levels. At the macro level, Sherman [21] first applied DEA to health care to assess whether relevant resources are being effectively used through efficiency. On this basis, researchers have further expanded the measurement of the HSE. Research methods have also gradually evolved from traditional DEA models to more comprehensive and efficient DEA models. These include the multistage DEA model [22, 23], the Super-SBM model [24], the Bootstrap DEA model [25], the game-crossover DEA model [26], and the dynamic network DEA model [27]. Some studies have also used the Malmquist productivity index to deconstruct the HSE [28, 29]. At the micro level, Valdmanis [30] used the DEA model to analyze the performance of Michigan hospitals. Subsequently, scholars have extensively used DEA and its extensions to assess the efficiency of healthcare organizations such as hospitals [31–34].

Still, as research has progressed, the shortcomings of the DEA model—namely its inability to separate the influence of random error terms and to statistically test hypotheses about inefficiency—have become increasingly prominent [35]. Additionally, the DEA model assumes constant returns to scale and that all decision-making units (DMUs) operate under the same production technology. It also assumes that inputs and outputs can be measured accurately and that no external environmental factors that affect efficiency exist [36]. In contrast, the SFA model assumes that DMUs operate under a stochastic production or cost frontier, allowing for random noise and inefficiency. It also assumes that the inefficiency is independently and identically distributed across DMUs and that the error term in the production or cost function follows a specific distribution, often assumed to be half-normal or truncated normal [37].

On this basis, the parametric approach represented by the SFA model has also been used to evaluate HSE at a macro or micro level. At the macro level, scholars have utilized the SFA model with different specifications to assess the HSE among different countries. For example, Kinfu [38] measured the HSE of the South African health system using the specifications of Aigner et al. (1977) and Broeck et al. (1977). De Cos [39] assessed the HSE in 29 OECD countries based on the specification of Schmidt and Sickles (1984). Ogloblin [40] evaluated the HSE in 78 countries using the specifications of Battese and Coelli (1995). In addition, several scholars have assessed the HSE using a true random or fixed-effects model [41, 42]. However, only a few studies have applied SFA to assess the HSE in China. Wen et al. [43] used the specifications of Battese and Coelli (1995) to assess the performance of the HSE in 31 provinces in China from 2009 to 2018. Li et al. [44] used the same methodology to evaluate regional HSE in China between 2009 and 2018. Kang et al. [45] compared the HSE between China and the Association of Southeast Asian Nations countries from 2015 to 2020 based on the specifications of Aigner et al. (1977) and Broeck et al. (1977). At the micro level, owing to the vital role of hospitals in the healthcare system, studies have increasingly used SFA to evaluate various efficiencies in hospitals [46–48]. However, few studies have utilized SFA to measure hospital efficiency in China. Xu et al. [49] investigated 50 public hospitals in the Chinese city of Beijing using the specifications of Battese and Coelli (1995). Chen et al. [50] introduced a Bayesian SFA model to assess the cost efficiency of Chinese provincial hospitals. Wei et al. [51] constructed a hospital-level fixed-effects stochastic frontier model and 5-year panel data from 89 hospitals to study the cost efficiency of Chinese hospitals. In addition, several scholars have evaluated the efficiency of healthcare systems and healthcare organizations using econometric models as well as functional models of production, cost, and distance. Evans et al. [52] estimated the HSE of 191 national health systems based on econometric modeling. Zhang et al. [53] measured the efficiency of China's healthcare delivery system in 2010 using the Cobb–Douglas production function. Shen [54] assessed the technical efficiency of operating hospitals in China from 2009 to 2016 using an aggregated directional distance function. Li [55] analyzed the cost efficiency of Washington State hospitals using a stochastic frontier Leontief cost function.

### Research on the temporal-spatial characteristics of the HSE

Little research has been done on the temporal-spatial performance of the HSE, and most research has been based on methods such as the spatial econometric model. Piedra [56] confirmed the existence of a positive spatial effect between the efficiency of public hospitals in Ecuador. Longo [57] investigated whether hospitals in the UK National Health Service improved the efficiency of adjacent hospitals and found that there was no spatial effect between hospitals in terms of efficiency. Mard [58] studied the impact of socioeconomic and demographic factors on health efficiency among Tunisian governorates and found that education had a positive influence on the efficiency of the healthcare system in both the region and its neighboring areas. However, the impact of unemployment was negative. Felder [59] confirmed that the HSE at the regional level in Germany was influenced by spatial dependence. Cavalieri [60] found that regional and institutional factors were more likely to affect heterogeneity in hospital efficiency across Italy than proximity effects.

Few studies have investigated the temporal-spatial characteristics of the HSE by applying quantitative methods such as kernel density estimation, spatial autocorrelation analysis methods, and Markov chain models. Li et al. [61] combined data envelopment analysis, kernel density estimation, and other methods to investigate the temporal-spatial characteristic of rural HSE in 29 regions of China from 2004 to 2018. The study found that China's rural HSE is growing slowly and that the inter-regional HSE shows a spatial pattern of "high center, low west." Wang et al. [18] used the Super-SBM model to estimate the HSE of 284 Chinese cities from 2009 to 2019 and explored the spillover effects and dynamic transfer trends of urban HSE with the Markov chain model. The results indicate that high potential exists for a shift from intermediate to high levels of HSE in each location. Urban HSE exhibits a progressively decreasing spatial layout with urban agglomeration cities at its core. Wu et al. [62] collected panel data from 2010 to 2019 to calculate the efficiency of primary healthcare services in central and western regions through the Super-SBM model, and they further analyzed the temporal-spatial variation patterns in efficiency by employing kernel density estimation and the Markov chain. They found an aggregation effect for both high and low primary healthcare services efficiencies in the study, which makes it difficult to achieve significant efficiency improvement in the short term. Chen et al. [63] measured the imbalance of health service supply in 31 provinces of China from 2005 to 2020 by the modified entropy method and investigated the temporal-spatial evolution trends of the health service supply capacity by using kernel density estimation and Markov chain. Studies have shown that the health service supply capacity in China is on an upward trend. However, the health service supply capacity is characterized by spatial imbalances, with the east being higher than the center.

By combining the above literature, we identified that the following deficiencies still exist in the study of the HSE. First, research on the HSE has focused on specific types or individual cities at the institutional level, i.e., with the institution as the unit of analysis. However, relatively few studies have been conducted on the assessment of the HSE performance at the system level on a national scale [64]. Second, most scholars have preferred to use the DEA models and their extended forms to study the HSE, while few scholars have used econometric models and functional models such as production, distance, and cost for their studies. However, the DEA model assumes no random errors and attributes all deviations from the efficient frontier to be caused by inefficiencies [65]. Further, models based on functions and econometrics are deterministic without sufficient consideration of random disturbances in the data. Moreover, related studies have employed the SFA model mainly from a static perspective and have not adequately considered the sources of dynamic changes in efficiency. Finally, existing studies have mainly examined the spatial effects of the HSE and its influencing factors only from a unitary perspective of time or space, and few studies have comprehensively and systematically discussed the temporal-spatial evolution process of the HSE [66, 67]. More importantly, the absence of a reasonable temporal-spatial analysis method usually leads to less comprehensive analysis and less accurate results [68].

Aiming at the deficiency of existing research, we made improvements in the following dimensions. First, regarding the research methodology, the main drawback of the DEA model is its assumption that there is no inefficiency measurement error or statistical noise. These statistical errors can lead to inaccurate efficiency scores and potentially misrepresent the true efficiency levels of DMUs. However, the SFA model deals with the effect of statistical noise (e.g., measurement error) and inefficiency error terms (i.e., inefficiency error that is controllable but does not reach the optimal part of the DMUs) on the results by explicitly assuming the form of the frontier production function and the distribution of the inefficiency term [69]. Moreover, as a benchmark or boundary method, SFA can effectively estimate the production level of DMUs considering the random perturbation of data [70]. Thus, this study evaluated China's macro-level HSE using the SFA model with a parametric approach, which improves the accuracy of the efficiency assessment. Second, we adopted the Malmquist index, a nonparametric model proposed by Caves [71], to overcome the shortcomings of SFA in capturing the source of productivity change and understand the drivers behind these changes. Finally, we simultaneously applied three quantitative analysis methods, namely, kernel density estimation, Markov chain, and spatial autocorrelation analysis, to deeply investigate the temporal-spatial correlation features of China's HSE and its evolutionary pattern. The reason we used all three methods together is that, first, the kernel density estimation method provides a temporal series static analysis of the overall level of regional efficiency. Second, the Markov chain model allows for further analyses of the trends in time-series dynamic shifts in efficiency levels from the perspective of provinces independently. These two approaches supplement each other considerably from the overall to the local context. Finally, geospatial factors were introduced into the study through exploratory spatial data analysis methodology to investigate the spatial evolution of China's HSE. These three approaches intertwine and complement each other, making our study more comprehensive.

## Methods and materials

### Efficiency measurement

#### Stochastic frontier analysis

Traditional production functions assume that producers obtain the greatest output with the fewest inputs; however, not all producers successfully overcome optimization issues [72]. To address this challenge, Aigner and Schmidt (1977) and Meeusen and Broeck (1977) [73, 74] developed the SFA model, which has had a major impact on productive econometric modeling and assessing the technical efficiency of firms. Later, scholars such as Battese [75, 76] refined the SFA model, mainly in terms of the treatment of inefficiencies and the application of panel data. Moreover, owing to the extensive research period in this paper, it is difficult to guarantee complete stability of individual efficiency. Furthermore, considering that the inefficient part of the HSE may change over time, we conducted our study based on the time-varying random-effects frontier model proposed by Battese et al. (1992) [77], which is given below.

1 $${Y}_{it}=f\left({x}_{it};\beta\,\right)\mathrm{exp}\left({v}_{it}-{u}_{it}\right),\,where\,i=\mathrm{1,2},\dots\,,n$$

In Eq. (1), ${Y}_{it}$ is the output; $f\left({x}_{it};\beta \right)$ is the production function; ${x}_{i}$ is the input factor vector, which is the vector of its coefficients to be estimated; ${v}_{it}$ is used to represent the effect of statistical error and various stochastic factors on the frontier output; and ${u}_{it}\ge 0$ is a time-varying technical inefficiency term that measures the relative production efficiency level and is independent of ${v}_{it}$.

Further, we quantitatively describe the effect of the timing factor on the inefficiency term ${u}_{it}$.

2 $$\begin{array}{c}{u}_{it}=\beta \left(t\right)\times {u}_{i}, i=\mathrm{1,2},3,\dots ,n\\ \beta \left(t\right)=exp\left\{-\eta \times \left(t-T\right)\right\}\end{array}$$

In Eq. (2), $T$ is the total periods used in the study, and $\eta$ is a time-varying coefficient to be estimated, reflecting the magnitude of the rate of change in technical efficiency. If $\eta <0$, $\beta \left(t\right)$ increases with $t$, and technical efficiency decreases; if $\eta >0$, $\beta \left(t\right)$ decreases with $t$ and technical efficiency improves; and if $\eta =0$, $\beta \left(t\right)$ does not change over time, and the technical efficiency does not change either.

Finally, owing to technical inefficiency and random noise, it is challenging for producers to achieve the frontier level of the production function. A producer’s technical efficiency (TE) is expressed by the ratio of the expectation of the producer’s output in the sample to the expectation of the stochastic frontier, as shown in Eq. (3).

3 $${TE}_{it}=\frac{E\left[f\left({x}_{it},\beta\,\right)exp\left({v}_{it}-{u}_{it}\right)\right]}{E\left(f\left({x}_{it},\beta \right)exp\left({v}_{it}-{u}_{it}\right)|{u}_{it}=0\right)}=exp\left\{-{\mu\,}_{it}\right\},\,where\,i=\mathrm{1,2},3,\dots\,,n$$

#### Production function setting for the SFA model

Following the study of Coelli et al. [78], we specify two forms of production function in the SFA model (i.e., Cobb-Douglass or Translog functional forms). However, researchers have different views on the usage of these two functions [79, 80].

First, the logarithmic expression of the Cobb–Douglas functional form is as follows:

4 $$Ln{Y}_{it}={\beta }_{0}+\sum_{j=1}^{N}{\beta\,}_{i}Ln{x}_{j,it}+{v}_{it}-{\mu }_{it}$$

In Eq. (4), ${Y}_{it}$ represents the output of healthcare services for $i$ DMUs in period $t$; $j$ represents the number of independent variables; ${x}_{j,it}$ indicates the corresponding input level of the ith decision unit at time $t$; ${\beta }_{0}$ is the constant term intercept; ${\beta }_{i}$ is the parameter to be estimated; and ${v}_{it}$ and ${u}_{it}$ are the same as in Eq. (1).

Second, the Translog function provides a second-order approximation that is a flexible functional form. Thus, the Translog function is also known as the Cobb–Douglas extended form, and the expression is as shown below.

5 $$Ln{Y}_{it}={\beta\,}_{0}+\sum_{j=1}^{N}{\beta }_{i}Ln{x}_{j,it}+\frac{1}{2}{\sum }_{j=1}^{N}{\sum }_{h=1}^{N}{\beta }_{jh}Ln{x}_{j,it}Ln{x}_{h,it}+{v}_{it}-{\mu }_{it}$$

Finally, we adjusted the form of Cobb–Douglas according to the current research purpose; the Cobb–Douglas production function of China's healthcare services can be expressed as below.

6 $$Ln{HSO}_{it}={\beta }_{0}+{\beta }_{1}Ln{L}_{it}+{\beta }_{2}Ln{K}_{it}+{v}_{it}-{\mu }_{it}, where i=\mathrm{1,2.3},\dots n$$

In Eq. (6), ${HSO}_{it}$ expresses the healthcare service output of the ith decision unit at time $t$; ${L}_{it}$ denotes the corresponding labor input of the ith decision unit at time $t$. ${K}_{it}$ denotes the corresponding capital investment of the ith decision unit at time $t$. ${\beta }_{0}$ is the constant term intercept; ${\beta }_{1} \mathrm{and }{\beta }_{2}$ are the parameters to be estimated; and ${v}_{it}$ and ${u}_{it}$ are the same as in Eq. (1).

In addition, if we extend the logarithmic of the Cobb–Douglas functional form, we obtain the logarithmic of the Translog functional form for China's healthcare services:

7 $$\begin{array}{c}Ln{HSO}_{it}={\beta }_{0}+{\beta }_{1}Ln{L}_{it}+{\beta }_{2}Ln{K}_{it}+\frac{1}{2}{\beta }_{3}{\left(Ln{L}_{it}\right)}^{2}+\frac{1}{2}{\beta }_{4}{\left(Ln{K}_{it}\right)}^{2}\\ +{\beta }_{5}Ln{K}_{it}Ln{L}_{it}+{v}_{it}-{\mu }_{it}\end{array}$$

In Eq. (7), ${\beta }_{0}$ is the constant term intercept; ${\beta }_{1},{\beta }_{2},{\beta }_{3},{\beta }_{4}$, and ${\beta }_{5}$ are the parameters to be estimated; and the other variables have the same meaning as in Eq. (6).

Notably, the Cobb–Douglas function has the advantage of having a simple model form with few parameters for easy estimation. However, it has the disadvantage of a fixed and constant factor elasticity of substitution. Although the Translog function overcomes this shortcoming, the Translog function is not necessarily superior to the Cobb–Douglas function. Thus, we followed the study of Kumbhakar et al. [81] to select the production function suitable for this study. The specific test steps are as follows:

8 $$LR=-2\left[Ln\left({H}_{0}\right)-Ln\left({H}_{1}\right)\right]$$

In Eq. (8), the original assumption is ${H}_{0}: {\beta }_{3}={\beta }_{4}={\beta }_{5}=0$ (i.e., the production function is assumed to be of the Cobb–Douglas form). Alternative assumption is ${H}_{1}:{\beta }_{3},{\beta }_{4},{\beta }_{5}\ne 0$ (i.e., the production function is assumed to be of the Translog form). $Ln\left({H}_{0}\right)$ represents the value of the log-likelihood function for the original hypothesis ${H}_{0}$. $Ln\left({H}_{1}\right)$ represents the value of the log-likelihood function of the alternative hypothesis ${H}_{1}$. Furthermore, the statistic obeys a mixed ${\lambda }^{2}$ distribution, provided the original hypothesis holds.

#### Parametric tests of the production function for the SFA model

To find suitable production function forms for the SFA model, first, we examined which functional form is most suitable for this study using Eq. (8). Second, we examined the distribution of technical inefficiency in the production function by utilizing the null hypothesis ${H}_{0}:\mu =0$. This null hypothesis assumes that healthcare providers are all operating on the technical efficiency frontier and that there are no asymmetric and random technical efficiencies in the inefficiency effects (i.e., technical inefficiencies have a half-normal distribution). If $\mu \ne 0$, then the technical inefficiency has a truncated normal distribution. Finally, we verified the presence of inefficiency by calculating the value of $\gamma$. The specific formula is as follows:

9 $$\gamma =\frac{{\delta }_{\mu }^{2}}{{\delta }_{\mu }^{2}+{\delta }_{v}^{2}}$$

In Eq. (9), $\gamma$ represents the magnitude of the variance of the inefficiency term; ${\delta }_{\mu }^{2}$ and ${\delta }_{v}^{2}$ represent the variances of the inefficiency and random shock terms, respectively. If $\gamma$ is close to 0, the statistical noise is perfectly correlated with the production variance. If $\gamma$ is close to 1, a significant portion of the error term comes from technical inefficiencies.

#### Malmquist index

The SFA model cannot capture the dynamic sources of variation in efficiency since it can only evaluate the static efficiency of each decision unit. Therefore, to examine the total factor productivity change in healthcare services in China, we used the Malmquist index (MI) model.

Total factor productivity (TFP) is a measure of the efficiency with which multiple inputs (such as labor and capital) are combined to produce output in a production process. It captures the portion of output growth that cannot be explained by changes in the quantities of inputs used [82]. Caves constructed the Malmquist productivity index in 1982 [71]. It is assumed that in each period $t=\mathrm{1,2},3,\dots ,T$, the production unit transforms the input ${e}^{t}$ into the output ${p}^{t}$ according to the production technology ${S}^{t}$. Then, the Malmquist index can be expressed by the distance function [83], i.e., ${D}_{O}^{t}=({p}^{t},{e}^{t})$. Based on this, the Malmquist index in period $t$ is defined below.

10 $${M}_{O}^{t}=\frac{{D}_{O}^{t}=({p}^{t+1},{e}^{t+1})}{{D}_{O}^{t}=({p}^{t},{e}^{t})}$$

Similarly, the Malmquist based on period $\text{t+1}$ can be expressed as below.

11 $${M}_{O}^{t+1}=\frac{{D}_{O}^{t+1}=({p}^{t+1},{e}^{t+1})}{{D}_{O}^{t+1}=({p}^{t},{e}^{t})}$$

According to Grosskopf et al. [84] and Färe et al. [85], the Malmquist index used in this study is the geometric mean of the two indices mentioned above, which avoids arbitrariness in the choice of the base period. The formula is shown as follows:

12 $${Malmquist\,Index=M}_{O}^{t}\left({p}^{t+1},{e}^{t+1},{p}^{t},{e}^{t}\right)\,{ = \left[\left(\frac{{D}_{O}^{t}=\left({p}^{t+1},{e}^{t+1}\right)}{{D}_{O}^{t}=\left({p}^{t},{e}^{t}\right)}\right)\left(\frac{{D}_{O}^{t+1}=\left({p}^{t+1},{e}^{t+1}\right)}{{D}_{O}^{t+1}=\left({p}^{t},{e}^{t}\right)}\right)\right]}^\frac{1}{2}$$

In Eq. (12), if $\text{MI > 1}$, it denotes a relative growth in TFP. If $\text{MI < 1}$, it denotes a relative decrease in TFP. If $\text{MI = 1}$, it indicates no change in TFP.

Accordingly, the Malmquist index can be divided into the two components below.

13 $$\begin{array}{cc}Malmquist Index&\,\begin{array}{cc}=&\,{M}_{o}^{t} \left({p}^{t+1},{e}^{t+1},{p}^{t},{e}^{t}\right)\end{array}\\ &\,\begin{array}{cc}=&\,\frac{{D}_{O}^{t+1}\left({p}^{t+1},{e}^{t+1}\right)}{{D}_{O}^{t+1}\left({p}^{t},{e}^{t}\right)}{\left[\left(\frac{{D}_{O}^{t}\left({p}^{t+1},{e}^{t+1}\right)}{{D}_{O}^{t+1}\left({p}^{t+1},{e}^{t+1}\right)}\right)\left(\frac{{D}_{O}^{t}\left({p}^{t},{e}^{t}\right)}{{D}_{O}^{t+1}\left({p}^{t},{e}^{t}\right)}\right)\right]}^\frac{1}{2}\end{array}\\\,&\,\begin{array}{cc}=&\,techch\times\,effch=techch\times\,\left(pech\times sech\right)\end{array}\end{array}$$

In Eq. (13), the MI decomposes the TFP change (tfpch) into two components: technical change (techch) and technical efficiency change (effch) [86]. effch denotes the level of improvement resulting from the technological innovation that occurred between period ${\text{t}}$ and period $\text{t+1}$ [87]. techch is used to evaluate the impact of changes on production fronts as they are sent. Further, effch comprises two components: pure efficiency (pech) and scale efficiency (sech). Pech estimates the managerial efficiency of DMUs, while sech estimates the appropriateness of the scale of DMUs.

### Time-evolution analysis

#### Kernel density estimation

To reflect the information on the distribution pattern and extension of the HSE in China, we used kernel density for estimation in this paper. Kernel density estimation is a nonparametric approach for kernel estimation. In this method, discrete variables are connected by smooth curves, while the distribution of random variables is represented by sequential of density curves [88, 89]. The basic principle is to assume that the random variables ${X}_{1},{X}_{2},\dots ,{X}_{N}$ are independently and identically distributed. Their density functions are denoted by $f\left(x\right)$, and the kernel density is estimated as shown below.

14 $$f\left(x\right)=\frac{1}{nh}\sum_{i=1}^{n}K\left(\frac{{X}_{i}-x}{h}\right)$$

15 $$K\left(x\right)=\frac{1}{\sqrt{2\pi }}exp\left(-\frac{{x}^{2}}{2}\right)$$

In Eq. (14), $n$ is the number of Chinese provinces studied in this work; ${X}_{i}$ is the observed value, i.e., the HSE of each province, and $x$ is the average of the observed values; $K\left(\bullet \right)$ is a Gaussian kernel function, which can be represented by Eq. (15); and $h$ is the bandwidth, which determines how accurately the kernel density curve is estimated. In this paper, by referring to Sliverman [90] approach, we set the bandwidth as below.

16 $$h=1.06\delta {N}^{-\frac{1}{5}}$$

In Eq. (16), $\delta$ is estimated by $min\left\{s,Q/1.34\right\}$, $s$ is the sample standard deviation, and $Q$ is the interquartile spacing.

#### Markov chain

While kernel density estimation can capture the distribution pattern of HSE over time as a whole, it does not deeply reflect the intrinsic dynamic trends and characteristics of the distribution. To address this challenge, a possible approach is to explore the internal dynamic trends of HSE in each region by estimating the transition probability matrix associated with Markov chains [91].

Markov chains are models of stochastic processes proposed by the Russian mathematician Markov. Markov chains treat random variables as discrete states and can be used to examine the mobility of the internal distribution between levels by calculating the probability of shifting the variables up or down a level after a time change through maximum likelihood estimation [92]. Thus, we adopted a Markov chain model to construct the state transfer probability matrix to meticulously grasp the relative state changes between high and low Chinese HSE at a specified time, as well as the possibility of changes. The specific steps are as follows:

A Markov chain behaves as a stochastic process $\left\{X\left(t\right),t\in T\right\}$, where the set $T$ of indices in this stochastic process corresponds to the individual periods. Then, for all periods $t$ and all possible states $i$, $j$, they satisfy the condition of Eq. (17).

Equation (17) indicates that the state of the random variable $X$ at period $t-1$ determines the probability of it being in state $j$ during period $t$.

17 $$\begin{array}{c}P=\left\{X\left(t\right)=\left.j\right|X\left(t-1\right)=i,X\left(t-2\right)={i}_{t-2},\dots ,X\left(0\right)={i}_{0}\right\}\\ =X\left(t\right)=\left.j\right| X\left(t-1\right)=i\end{array}$$

Let $X\left(t\right)=j$; in other words, $j$ represents the state in period $t$, while ${P}_{ij}$ denotes the probability of all the transitional probabilities. Here, we classify the HSE of provinces into four types based on the quartiles (0.25, 0.5, and 0.75 division points), with the types indicated as $K=1, \mathrm{2,3},4$. The traditional Markov dynamic transformation probability matrix is illustrated in Table 1.

**Table 1:** Traditional Markov dynamic transformation probability matrix

${}^{{t}_{i}}\!\left/ \!{}_{{t}_{i+1}}\right.$	1	2	3	4
1	${P}_{11}$	${P}_{12}$	${P}_{13}$	${P}_{14}$
2	${P}_{21}$	${P}_{22}$	${P}_{23}$	${P}_{24}$
3	${P}_{31}$	${P}_{32}$	${P}_{33}$	${P}_{34}$
4	${P}_{41}$	${P}_{42}$	${P}_{43}$	${P}_{44}$

In Table 1, ${P}_{ij}$ means the state transfer probability that the HSE in period $t$ is of state $i$ and shifts to state $j$ in period $t+1$. It can be expressed as ${P}_{ij}$=${n}_{ij}/{n}_{i}$, where ${n}_{ij}$ indicates the summation of the number of districts in state $i$ in period $t$, and the count of districts transfers to state $j$ in period $t+1$; ${n}_{i}$ means the summation of the number of districts with state $j$ in all years. Then, we can construct a $4\times 4$ transfer probability matrix by categorizing the HSE into four kinds. Thus, we can analyze the dynamic transition trend and evolutionary pattern of the HSE in China and identify whether the dynamic transition trend is upward, downward, or unchanged.

Note that the traditional Markov chains still do not consider the effect of geographic and spatial factors on HSE transfer in China. Thus, to further analyze the spatial transfer trends of the HSE in 31 provinces in China, in addition to adding spatial lags to the traditional Markov transfer chain, we also constructed a spatial Markov transfer matrix using the distance between neighboring provinces as the weight of spatial lags to explore the relationship between the dynamic transition probabilities of HSE in the province and neighboring provinces. The specific step is to include the corresponding spatial lag term in the traditional Markov dynamic transformation probability matrix $(K\times K)$ and expand it to the spatial Markov dynamic transformation probability matrix $(K\times K\dots \times K)$ [93]. As shown in Table 2, we extended the traditional Markov dynamic transformation probability matrix $(4\times 4)$ of this study to a spatial Markov dynamic transformation probability matrix $(4\times 4\times 4\times 4)$, which presents the probabilities of provinces transitioning from state $i$ to state $j$ over the study period, considering the spatial lag terms $\mathrm{I},\mathrm{II},\mathrm{III},$ and $\mathrm{IV}$.

**Table 2:** Spatial Markov dynamic transformation probability matrix

Spatial lag term categories	${}^{{t}_{i}}\!\left/ \!{}_{{t}_{i+1}}\right.$	1	2	3	4
$\mathrm{I}$	1	${P}_{{}^{11}\!\left/ \!{}_{1}\right.}$	${P}_{{}^{12}\!\left/ \!{}_{1}\right.}$	${P}_{{}^{13}\!\left/ \!{}_{1}\right.}$	${P}_{{}^{14}\!\left/ \!{}_{1}\right.}$
	2	${P}_{{}^{21}\!\left/ \!{}_{1}\right.}$	${P}_{{}^{22}\!\left/ \!{}_{1}\right.}$	${P}_{{}^{23}\!\left/ \!{}_{1}\right.}$	${P}_{{}^{24}\!\left/ \!{}_{1}\right.}$
	3	${P}_{{}^{31}\!\left/ \!{}_{1}\right.}$	${P}_{{}^{32}\!\left/ \!{}_{1}\right.}$	${P}_{{}^{33}\!\left/ \!{}_{1}\right.}$	${P}_{{}^{34}\!\left/ \!{}_{1}\right.}$
	4	${P}_{{}^{41}\!\left/ \!{}_{1}\right.}$	${P}_{{}^{42}\!\left/ \!{}_{1}\right.}$	${P}_{{}^{43}\!\left/ \!{}_{1}\right.}$	${P}_{{}^{44}\!\left/ \!{}_{1}\right.}$
$\mathrm{II}$	1	${P}_{{}^{11}\!\left/ \!{}_{2}\right.}$	${P}_{{}^{12}\!\left/ \!{}_{2}\right.}$	${P}_{{}^{13}\!\left/ \!{}_{2}\right.}$	${P}_{{}^{14}\!\left/ \!{}_{2}\right.}$
	2	${P}_{{}^{21}\!\left/ \!{}_{2}\right.}$	${P}_{{}^{22}\!\left/ \!{}_{2}\right.}$	${P}_{{}^{23}\!\left/ \!{}_{2}\right.}$	${P}_{{}^{24}\!\left/ \!{}_{2}\right.}$
	3	${P}_{{}^{31}\!\left/ \!{}_{2}\right.}$	${P}_{{}^{32}\!\left/ \!{}_{2}\right.}$	${P}_{{}^{33}\!\left/ \!{}_{2}\right.}$	${P}_{{}^{34}\!\left/ \!{}_{2}\right.}$
	4	${P}_{{}^{41}\!\left/ \!{}_{2}\right.}$	${P}_{{}^{42}\!\left/ \!{}_{2}\right.}$	${P}_{{}^{43}\!\left/ \!{}_{2}\right.}$	${P}_{{}^{44}\!\left/ \!{}_{2}\right.}$
$\mathrm{III}$	1	${P}_{{}^{11}\!\left/ \!{}_{3}\right.}$	${P}_{{}^{12}\!\left/ \!{}_{3}\right.}$	${P}_{{}^{13}\!\left/ \!{}_{3}\right.}$	${P}_{{}^{14}\!\left/ \!{}_{3}\right.}$
	2	${P}_{{}^{21}\!\left/ \!{}_{3}\right.}$	${P}_{{}^{22}\!\left/ \!{}_{3}\right.}$	${P}_{{}^{23}\!\left/ \!{}_{3}\right.}$	${P}_{{}^{24}\!\left/ \!{}_{3}\right.}$
	3	${P}_{{}^{31}\!\left/ \!{}_{3}\right.}$	${P}_{{}^{32}\!\left/ \!{}_{3}\right.}$	${P}_{{}^{33}\!\left/ \!{}_{3}\right.}$	${P}_{{}^{34}\!\left/ \!{}_{3}\right.}$
	4	${P}_{{}^{41}\!\left/ \!{}_{3}\right.}$	${P}_{{}^{42}\!\left/ \!{}_{3}\right.}$	${P}_{{}^{43}\!\left/ \!{}_{3}\right.}$	${P}_{{}^{44}\!\left/ \!{}_{3}\right.}$
$\mathrm{IV}$	1	${P}_{{}^{11}\!\left/ \!{}_{4}\right.}$	${P}_{{}^{12}\!\left/ \!{}_{4}\right.}$	${P}_{{}^{13}\!\left/ \!{}_{4}\right.}$	${P}_{{}^{14}\!\left/ \!{}_{4}\right.}$
	2	${P}_{{}^{21}\!\left/ \!{}_{4}\right.}$	${P}_{{}^{22}\!\left/ \!{}_{4}\right.}$	${P}_{{}^{23}\!\left/ \!{}_{4}\right.}$	${P}_{{}^{24}\!\left/ \!{}_{4}\right.}$
	3	${P}_{{}^{31}\!\left/ \!{}_{4}\right.}$	${P}_{{}^{32}\!\left/ \!{}_{4}\right.}$	${P}_{{}^{33}\!\left/ \!{}_{4}\right.}$	${P}_{{}^{34}\!\left/ \!{}_{4}\right.}$
	4	${P}_{{}^{41}\!\left/ \!{}_{4}\right.}$	${P}_{{}^{42}\!\left/ \!{}_{4}\right.}$	${P}_{{}^{43}\!\left/ \!{}_{4}\right.}$	${P}_{{}^{44}\!\left/ \!{}_{4}\right.}$

### Spatial-evolution analysis

#### Exploratory spatial data analysis (ESDA)

ESDA is a spatial econometric methodology for studying the associations of a phenomenon in geospatial space with the values of attributes in neighboring elements [94]. As shown in Table 3, ESDA primarily consists of the global spatial autocorrelation analysis and local spatial autocorrelation analysis, which are analyses of possible interdependencies and correlations between certain observations in a specific range [95]. Thus, this study applied the ESDA model to comprehensively reveal the overall spatial autocorrelation and internal agglomeration features of the HSE in China.

**Table 3:** Exploratory spatial data analysis (ESDA)

Methodology	Purpose	Output Format
Global autocorrelation	Determine spatial correlation and detect spatial heterogeneity	Spatial correlation Index
Local autocorrelation	Determine the degree of aggregation and identify spatial distribution patterns	Graphical display of spatial clustering patterns

#### Global autocorrelation

The global Moran’s I index was used to check for correlations in the HSE in each province before doing the spatial correlation study. Moran’s I index is defined as follows:

18 $$\mathrm M\mathrm o\mathrm r\mathrm a\mathrm n'\mathrm s\,\mathrm I=\frac{\sum_{i=1}^n\sum_{j=1}^nW_{ij}\left(y_i-\overline y\right)\left(y_j-\overline y\right)}{\sum_{i=1}^n\left(y_i-\overline y\right)^2}\times\frac n{\sum_{i=1}^n\sum_{j=1}^nW_{ij}}$$

In Eq. (18), $n$ is the subject of the study (i.e., the overall number of provinces); ${y}_{i}$ and ${y}_{j}$ represent the HSE of the $i$ province and $j$ province, respectively; $\overline{y }$ is the mean value of ${y}_{i}$ and ${y}_{j}$, and ${W}_{ij}$ is the neighboring space weight matrix. Moran’s I index takes values in the range of $\left[-\mathrm{1,1}\right]$. If the variable is positively correlated in space (Moran’s I > 0), provinces with higher (or lower) levels of HSE are more spatially clustered; if the variable is negatively correlated in space (Moran’s I < 0), there are spatial differences in the HSE between each province and its neighboring provinces. If the variable is spatially constant (Moran’s I = 0), then each province’s HSE randomly distributes in each province in space [96].

#### Local autocorrelation

Local spatial autocorrelation analysis was used to analyze the spatial correlation between each spatial object and its neighboring units in a specific region to reflect the local characteristic differences in the distribution of spatial objects. It can overcome the global spatial autocorrelation analysis, which only considers a single value to describe the degree of spatial correlation between the items under study and disregards any possible instability defects in the local area [97]. Therefore, the local Moran’s I reveals the spatial clustering state of efficiency between a province and its neighboring provinces. Among them, the H–H type represents the high-value region of the HSE surrounded by high-value adjacent units; the H–L type represents the high-value region surrounded by low-value adjacent units; the L-L type represents the low-value region surrounded by low-value adjacent units; and the L–H type represents the low-value region surrounded by high-value adjacent units.

### Indicator selection

#### Health service input indicators

Most investments in healthcare services are measured in terms of human, financial, and material resources [98]. However, Grossman (1972) brought up that the financial investment in health services frequently covers the investment in hiring different types of technicians and the investment in purchasing medical equipment, which can cause double counting issues with the current input indicators. As a result, the input indicators only calculate human and material capital input. In related studies, the number of health personnel, number of healthcare technicians, number of registered nurses, and number of licensed (assistant) doctors have usually been selected as inputs of human capital [99–101], while the number of beds, number of health facilities, and more than 10,000 yuan of equipment have been used as inputs for material resources [102, 103]. Therefore, this study selected the number of health technicians per 1,000 population and the number of beds in medical and health institutions per 1,000 population as human capital input and material capital input, respectively.

#### Health service output indicators

In terms of output indicators, owing to the complexity and particularity of the medical and health industry, its output is usually measured by disease cures and improvement of people’s health, but these are difficult to quantify. Associated studies have used the number of emergency and outpatient visits, bed utilization, admissions (discharges), and surgical inpatient visits as quantitative indicators of the supply of healthcare services to reflect the output of healthcare resources [104–106]. As a result, we chose the number of outpatient and emergency department visits, hospital discharges, and surgical procedures performed in the hospital as output indicators. Since the standard SFA model is limited to a single output [107, 108], it is necessary to aggregate the number of outpatients and emergency department visits, hospital discharges, and surgical procedures performed in the hospital into a single variable. Thus, referring to Xu et al. [109], principal component analysis was used to logarithmically transform and weight the three indicators to create the output index, as each of the output indicators has its own bias, thus maximizing the amount of information that could be found in the output indicators.

Furthermore, to control the effect of heteroskedasticity and ensure the smoothness of the data, all variables in this study were logarithmically transformed. The specific indicators and descriptive statistics of the input and output variables are presented in Table 4. The table shows that, from 2010 to 2020, the number of health technicians per 1,000 population and the number of beds in medical and health institutions per 1,000 population in China trended upward each year. The output index after transformation with principal component analysis and the number of outpatient and emergency department visits, hospital discharges, and surgical procedures performed in the hospital increased every year from 2010 to 2019, then dropped slowly from 2019 to 2020.

**Table 4:** Specific indicators and descriptive statistics of the input and output variables from 2010–2020

Period		Input indicators (unit)		Output indicators (unit)			
		Number of health technicians per 1,000 population (Person)	Number of beds in medical and health institutions per 1,000 population (bed)	Number of surgical procedures performed in the hospital (Persons)	Number of outpatient and emergency department visits (Person-times)	Number of hospital discharges (Persons)	Output index after transformation with principal component analysis
2010	Mean	1.515	1.307	13.4	18.66	15	15.68
	Median	1.484	1.258	13.61	18.78	15.21	15.8
	SD	0.34	0.26	1.03	0.946	0.983	0.971
	Min	0.908	0.92	9.917	16.04	11.95	12.63
	Max	2.609	2.007	14.97	20.18	16.22	17.09
2011	Mean	1.562	1.37	13.52	18.73	15.08	15.77
	Median	1.562	1.335	13.73	18.89	15.3	15.9
	SD	0.333	0.242	1.025	0.949	0.978	0.969
	Min	0.986	1.019	10.17	16.09	11.99	12.74
	Max	2.653	2.022	15.12	20.26	16.3	17.19
2012	Mean	1.6	1.437	13.63	18.81	15.22	15.88
	Median	1.609	1.463	13.86	19	15.44	16.04
	SD	0.204	0.149	1.056	0.969	1.018	0.998
	Min	1.109	1.001	10.06	16.08	11.88	12.66
	Max	2.249	1.773	15.23	20.36	16.45	17.3
2013	Mean	1.694	1.514	13.72	18.88	15.3	15.96
	Median	1.696	1.554	13.9	19.05	15.51	16.13
	SD	0.311	0.131	1.022	0.961	0.989	0.974
	Min	1.292	1.261	10.37	16.23	12.2	12.92
	Max	2.738	1.802	15.29	20.42	16.48	17.35
2014	Mean	1.719	1.572	13.82	18.92	15.37	16.03
	Median	1.714	1.591	13.97	19.12	15.59	16.18
	SD	0.169	0.132	1.014	0.956	0.977	0.965
	Min	1.399	1.322	10.54	16.33	12.35	13.06
	Max	2.294	1.828	15.36	20.45	16.52	17.41
2015	Mean	1.767	1.627	13.85	18.93	15.4	16.05
	Median	1.758	1.637	13.98	19.11	15.64	16.2
	SD	0.165	0.125	1.024	0.956	0.964	0.964
	Min	1.482	1.391	10.57	16.36	12.57	13.16
	Max	2.342	1.852	15.42	20.46	16.55	17.45
2016	Mean	1.815	1.671	13.96	18.97	15.48	16.13
	Median	1.808	1.685	14.1	19.13	15.69	16.28
	SD	0.166	0.131	1.013	0.952	0.969	0.962
	Min	1.504	1.437	10.82	16.4	12.57	13.25
	Max	2.38	1.878	15.53	20.49	16.64	17.52
2017	Mean	1.872	1.733	14.05	19	15.55	16.19
	Median	1.841	1.744	14.17	19.14	15.8	16.34
	SD	0.159	0.135	1.013	0.943	0.969	0.958
	Min	1.589	1.479	10.95	16.52	12.7	13.38
	Max	2.425	1.924	15.66	20.52	16.72	17.59
2018	Mean	1.925	1.781	14.14	19.01	15.59	16.24
	Median	1.902	1.802	14.29	19.13	15.81	16.41
	SD	0.155	0.14	1.033	0.949	0.98	0.97
	Min	1.668	1.475	10.99	16.52	12.64	13.37
	Max	2.477	1.975	15.81	20.53	16.77	17.66
2019	Mean	1.984	1.821	14.25	19.06	15.63	16.31
	Median	1.96	1.845	14.41	19.24	15.83	16.44
	SD	0.149	0.142	1.035	0.958	0.986	0.976
	Min	1.74	1.475	11.08	16.52	12.63	13.4
	Max	2.534	2.02	15.94	20.59	16.82	17.74
2020	Mean	2.04	1.858	14.2	18.94	15.47	16.2
	Median	2.014	1.899	14.42	19.02	15.72	16.39
	SD	0.131	0.141	1.029	0.951	0.984	0.971
	Min	1.829	1.5	11.19	16.46	12.7	13.45
	Max	2.534	2.073	15.9	20.38	16.72	17.62

All variables are converted into logarithm

#### Data source

**Fig. 1:**
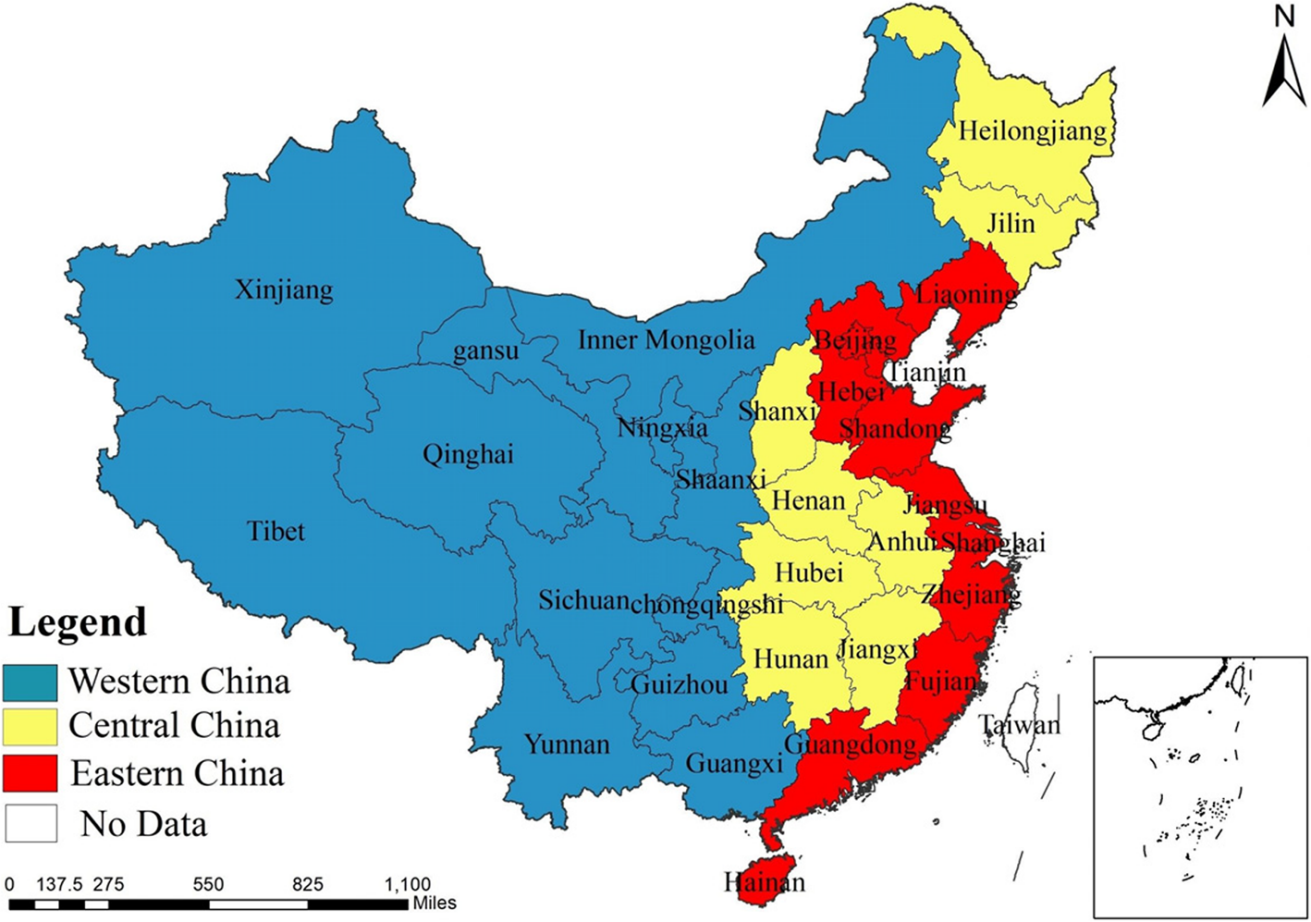
Distribution map of western, central, and eastern regions in China

The study’s data cover 31 provinces in China from 2010 to 2020, excluding Taiwan, Hong Kong, and Macao. We divided the provinces into regions based on geographical differences: eastern, central, and western (shown in Fig. 1). The eastern region includes Beijing, Tianjin, Hebei, Liaoning, Shanghai, Jiangsu, Zhejiang, Fujian, Shandong, Guangdong, and Hainan. The central part includes Shanxi, Jilin, Heilongjiang, Anhui, Jiangxi, Henan, Hubei, and Hunan. The western area includes Inner Mongolia, Chongqing, Guangxi, Sichuan, Guizhou, Yunnan, Tibet, Shaanxi, Gansu, Qinghai, Ningxia, and Xinjiang. Moreover, the data are from the “China Statistical Yearbook” and “China Health Statistical Yearbook”.

## Results of the empirical analysis

### Estimation results of the production function for the SFA model

We estimated the Cobb–Douglas and Translog production functions using maximum likelihood estimation. The results of the calculations are shown in Table 5. The table shows that the value of $LR$ is − 234.396, while the mixed ${\lambda }^{2}$ distribution at a 5% level of significance is ${\lambda }_{0.05}^{2}\left(3\right)=7.82$. Evidently, the $LR$ statistic is smaller than the critical value of the mixed ${\lambda }^{2}$ distribution. This implies that we should accept the original hypothesis that the Cobb–Douglas form is more appropriate for the production function of healthcare services in China. Furthermore, in terms of the elasticity of output for health technicians $\left({\beta }_{1}=0.303\right)$ and the elasticity of output for the number of beds in health facilities $\left({\beta }_{2}=0.128\right)$, for every 10% increase in the number of health technicians, the total output of health services increases by 3.03%. Similarly, for every 10% increase in the number of beds, the total output of health services increases by 1.28%.

**Table 5:** Estimation of stochastic frontier production function

parameter	Cobb–Douglas function	Translog function
${\beta }_{0}$	18.817***	14.309***
${\beta }_{1}$	0.303***	2.477***
${\beta }_{2}$	0.128	0.346
${\beta }_{3}$		0.505**
${\beta }_{4}$		1.601***
${\beta }_{5}$		-2.687***
${\delta }^{2}$	1.486***	0.499***
$\gamma$	0.995***	0.981***
$\eta$	0.014***	0.010***
$\mu$	2.433***	1.250***
Log Likelihood	232.519	115.321

^*^p < 0.05; **p < 0.01; ***p < 0.001

Further, $\mu =2.443 \left(p<0.01\right)$ in the Cobb–Douglas production function, which suggests that the truncated normal distribution is more appropriate for this study than the half-normal distribution in the distribution of technical inefficiency. Moreover, the value of $\gamma$ is 0.995 $\left(p<0.01\right)$, which shows that 99.5% of the random error term is associated with the inefficiency error term and that only 0.5% is associated with the statistical error. Further, the estimated parameter $\eta =0.014 \left(p<0.01\right)$ demonstrates that the effect of the time factor on $\beta \left(t\right)$ decreases at an increasing rate (i.e., the inefficiency component of the technical efficiency decreases over time, and the HSE increases). Thus, compared with the fixed-effects SFA model, which assumes that the inefficiency is time-invariant, the random-effects SFA model, assuming that the inefficiency is time-varying, is more suitable for this study [110].

Combined with the above analyses, we believe that a time-varying random-effects model truncating the normal distribution SFA model of the Cobb–Douglas form better fits the data in this study.

### Static assessment of China’s HSE

According to the model suitability and robustness tests, we calculated the efficiencies using selected input and output variables and data and using the time-varying random-effects model truncating the normal distribution SFA model of the Cobb–Douglas form through the Frontier 4.1 program. The results are shown in Table 6.

**Table 6:** Results of Chinese provincial HSE from 2010 to 2020

Prov	2010	2011	2012	2013	2014	2015	2016	2017	2018	2019	2020	Avg
Beijing	0.827	0.829	0.831	0.833	0.836	0.838	0.840	0.842	0.844	0.847	0.849	0.838
Tianjin	0.790	0.793	0.796	0.798	0.801	0.804	0.806	0.809	0.812	0.814	0.817	0.804
Hebei	0.869	0.871	0.873	0.874	0.876	0.878	0.879	0.881	0.883	0.884	0.886	0.878
Shanghai	0.839	0.841	0.843	0.845	0.847	0.850	0.852	0.854	0.856	0.858	0.859	0.849
Jiangsu	0.887	0.888	0.890	0.891	0.893	0.894	0.896	0.897	0.898	0.900	0.901	0.894
Zhejiang	0.879	0.881	0.882	0.884	0.886	0.887	0.889	0.890	0.892	0.893	0.895	0.887
Fujian	0.840	0.842	0.844	0.846	0.848	0.850	0.852	0.854	0.856	0.858	0.860	0.850
Shandong	0.895	0.897	0.898	0.899	0.901	0.902	0.903	0.905	0.906	0.907	0.909	0.902
Guangdong	0.912	0.913	0.914	0.915	0.917	0.918	0.919	0.920	0.921	0.922	0.923	0.918
Hainan	0.747	0.750	0.753	0.757	0.760	0.763	0.767	0.770	0.773	0.776	0.779	0.763
Liaoning	0.836	0.839	0.841	0.843	0.845	0.847	0.849	0.851	0.853	0.855	0.857	0.847
Eastern Region	0.847	0.849	0.851	0.853	0.855	0.857	0.859	0.861	0.863	0.865	0.867	0.857
Shanxi	0.817	0.819	0.822	0.824	0.826	0.829	0.831	0.833	0.836	0.838	0.840	0.829
Anhui	0.857	0.859	0.861	0.863	0.865	0.867	0.869	0.870	0.872	0.874	0.876	0.867
Jiangxi	0.844	0.846	0.848	0.850	0.852	0.854	0.856	0.858	0.860	0.862	0.864	0.854
Henan	0.890	0.891	0.893	0.894	0.896	0.897	0.898	0.900	0.901	0.903	0.904	0.897
Hubei	0.869	0.871	0.872	0.874	0.876	0.878	0.879	0.881	0.882	0.884	0.886	0.877
Hunan	0.865	0.867	0.868	0.870	0.872	0.874	0.875	0.877	0.879	0.880	0.882	0.874
Jilin	0.803	0.806	0.808	0.811	0.814	0.816	0.819	0.821	0.823	0.826	0.828	0.816
Heilongjiang	0.821	0.824	0.826	0.828	0.831	0.833	0.835	0.837	0.840	0.842	0.844	0.833
Central Region	0.846	0.848	0.850	0.852	0.854	0.856	0.858	0.860	0.862	0.864	0.865	0.856
Inner Mongolia	0.800	0.803	0.805	0.808	0.811	0.813	0.816	0.818	0.821	0.823	0.825	0.813
Chongqing	0.853	0.855	0.857	0.859	0.861	0.862	0.864	0.866	0.868	0.870	0.872	0.862
Sichuan	0.827	0.830	0.832	0.834	0.837	0.839	0.841	0.843	0.845	0.847	0.849	0.839
Guizhou	0.887	0.889	0.890	0.892	0.893	0.895	0.896	0.897	0.899	0.900	0.902	0.895
Yunnan	0.831	0.833	0.835	0.837	0.840	0.842	0.844	0.846	0.848	0.850	0.852	0.842
Shaanxi	0.851	0.853	0.855	0.857	0.859	0.860	0.862	0.864	0.866	0.868	0.870	0.860
Tibet	0.666	0.670	0.675	0.679	0.684	0.688	0.692	0.696	0.700	0.704	0.709	0.688
Gansu	0.839	0.841	0.843	0.845	0.847	0.850	0.852	0.854	0.856	0.858	0.859	0.849
Qinghai	0.805	0.807	0.810	0.813	0.815	0.818	0.820	0.822	0.825	0.827	0.830	0.817
Ningxia	0.721	0.725	0.729	0.733	0.736	0.740	0.743	0.747	0.750	0.754	0.757	0.740
Xinjiang	0.742	0.745	0.749	0.752	0.756	0.759	0.762	0.765	0.769	0.772	0.775	0.759
Guangxi	0.813	0.816	0.818	0.821	0.823	0.825	0.828	0.830	0.832	0.835	0.837	0.825
Western Region	0.803	0.806	0.808	0.811	0.813	0.816	0.818	0.821	0.823	0.826	0.828	0.816
Avg	0.830	0.832	0.834	0.836	0.839	0.841	0.843	0.845	0.847	0.849	0.851	0.841

As can be seen from Table 6, the overall average HSE from 2010 to 2020 is less than 1, ranging from 0.830 to 0.851, with an average yearly rise of 0.26%. This indicates that China’s total healthcare system efficiency can be improved.

**Fig. 2:**
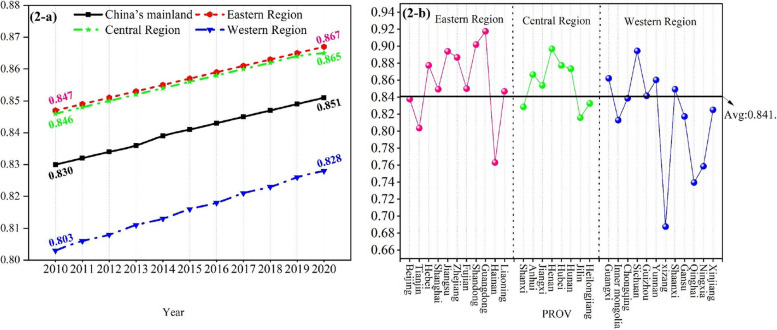
The healthcare service efficiency of different provinces and regions. **a** presents the average healthcare service efficiency (geometric mean) of China’s mainland and the three regions from 2010 to 2020; **b** shows the average healthcare service efficiency (geometric mean) in 31 provinces of China from 2010 to 2020

From an inter-regional perspective (shown in Fig. 2a), the HSE is heterogeneous across regions. The eastern region has the highest efficiency, while the western region has the lowest efficiency. In particular, the mean value of HSE in the eastern part varies from 0.847 to 0.867, with an average annual growth rate of 0.22%; in the central region, it varies from 0.846 to 0.865, with an average annual growth rate of 0.23%; and in the western region, it varies from 0.803 to 0.828, with an average annual growth rate of 0.31%. Generally, the eastern region’s efficiency level has always been at the top and has emerged as the “primary contributor” to the country’s efficiency. Provinces with poor efficiency, however, are primarily found in the west. This prevents the advancement of the Chinese healthcare system’s overall efficiency.

From an intra-regional perspective (shown in Fig. 2b), the HSE varies greatly between provinces, but the geographical classification does not reflect this intra-regional heterogeneity. For instance, in the eastern area, Guangdong’s efficiency is 0.918, whereas Hainan’s only averages 0.763. In the central region, Henan’s mean efficiency value is 0.897, whereas Jilin’s is only 0.816. Guizhou has an average efficiency score of 0.895 in the western region compared with Tibet’s 0.688. According to the analysis above, owing to variations in economic development and population distribution, different regions and provinces had different investment scales, allocation strategies, and management levels for medical and health resources, leading to spatial variations in the HSE.

### Dynamic assessment of China’s HSE

To further explore the source of dynamic changes in the HSE, we used DEAP2.1 software to measure the tfpch of healthcare services in China from 2010 to 2020. The outcomes are displayed in Table 7.

**Table 7:** The Malmquist index and its decomposition for China’s healthcare services from 2010 to 2020

Year	techch	effch	pech	sech	tfpch
2010–2011	1.038	1.013	1.034	0.98	1.051
2011–2012	1.076	1.02	1.04	0.981	1.098
2012–2013	1.002	1.031	1.065	0.968	1.033
2013–2014	1.017	1.01	1.017	0.993	1.027
2014–2015	1.002	0.999	1.015	0.984	1
2015–2016	1.042	1.003	0.995	1.008	1.045
2016–2017	1.045	0.991	1.006	0.986	1.036
2017–2018	1.034	0.982	1.002	0.98	1.015
2018–2019	1.038	0.979	0.999	0.979	1.016
2019–2020	0.886	0.95	0.999	0.951	0.842
Geometric mean	1.018	0.998	1.017	0.981	1.016

*tfpch* Total factor productivity change, *techch* Technical change, *effch* Technical efficiency chang, *pech* Pure efficiency, *sech* Scale efficiency

Overall, the average value of tfpch from 2010 to 2020 is 1.016, with an average growth rate of 1.6%. Nevertheless, the tfpch growth rate for healthcare services varies over time, with the largest increase of 9.8% occurring from 2011 to 2012 and the biggest decrease of 15.8% occurring from 2019 to 2020. This indicates that the change in tfpch for healthcare services has not been constant. From the perspective of decomposition indicators, the average values of pech and techch are both more than 1 (1.017 and 1.018), an increase of 1.7% and 1.8%, respectively. This indicates that both the pure efficiency change index and technology change index have shown an upward trend, which has jointly promoted the increase in TFP. The average values of sech and effch are both less than 1 (0.981 and 0.998), a decrease of 1.9% and 0.2%, respectively. The above analysis results indicate that there has been a small efficiency improvement owing to variables such as factor mix and management. Technical change may be considered the primary force behind developing the TFP of healthcare services in China.

In terms of time series, pech and effch increase by 3.4% and 1.3%, respectively, and sech decreases by 0.2% from 2010 to 2011. However, techch increases by 3.8%, causing tfpch to rise by 5.1%. From 2011 to 2014, sech decreases by 1.9%, 3.2%, and 0.7%; pech increases by 4%, 6.5%, and 1.7%, so effch increases by 2%, 3.1%, and 1%, respectively. Moreover, techch rises by 7.6%, 0.2%, and 1.7%, respectively, and finally tfpch shows an upward trend. From 2014 to 2019, sech and pech both fluctuate downward, so effch declines by 2.1% in 2018–2019, while techch and tfpch maintain an upward trend. Note that in 2019–2020, pech and sech decline by 0.1% and 4.9%, respectively, resulting in a 5% decline in effch, while techch declines by as much as 11.4%, and finally tfpch declines by 15.8%.

### Time series analysis of China’s HSE

**Fig. 3:**
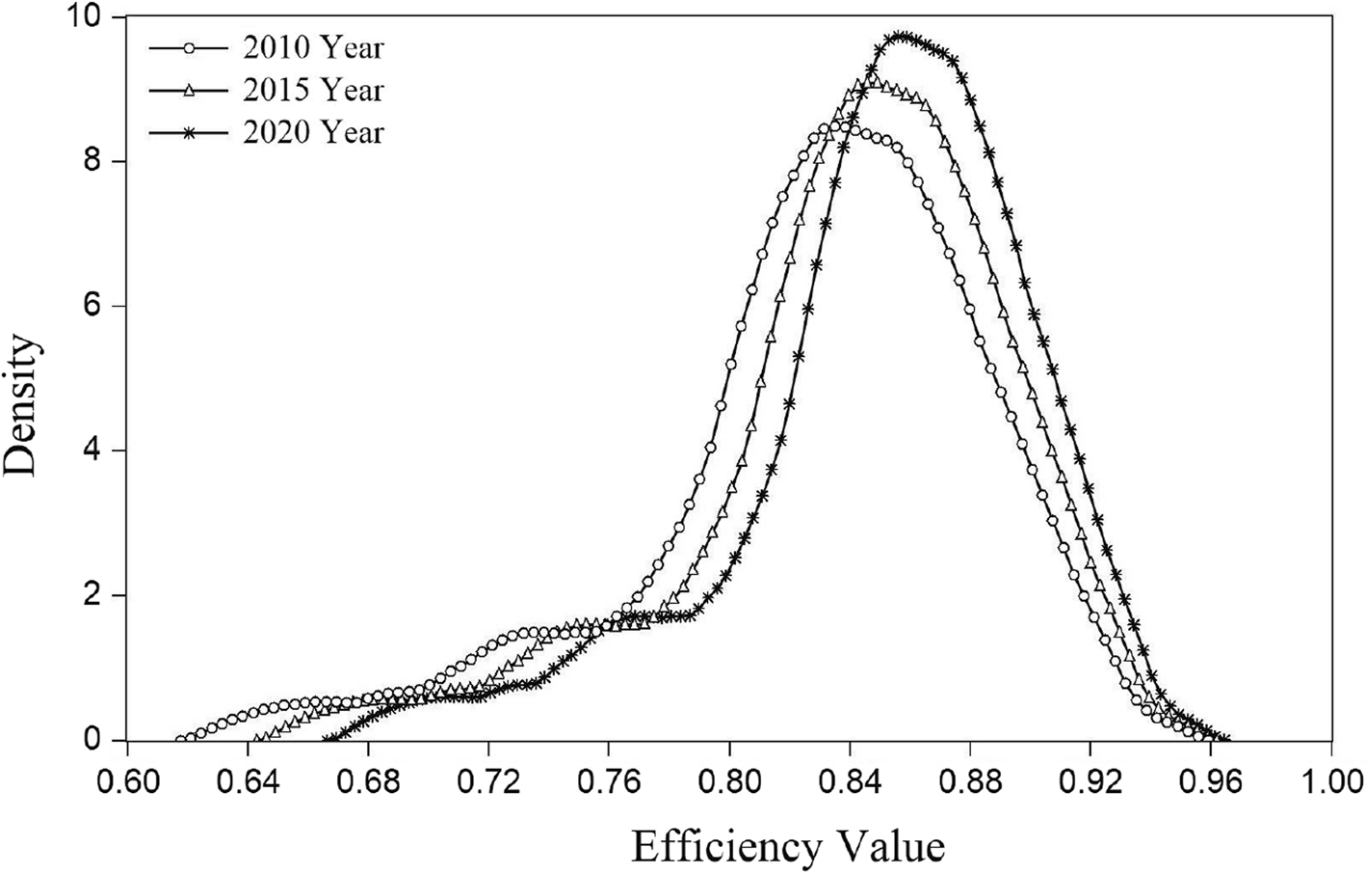
kernel density distribution of China's healthcare service efficiency from 2010 to 2020

By analyzing the results of measuring the HSE in China, we can only obtain simple static conclusions, not describe the dynamic transferred evolutionary trend and overall temporal series process of the efficiency. Thus, in this study, the efficiency values for 2010, 2015, and 2020 were selected at equal intervals, and the kernel density estimation function based on Gaussian normal distribution was applied to plot the kernel density distribution corresponding to them by Eviews 10 software (see Fig. 3). Accordingly, we analyzed the trends from the distribution location and shape. We identified the following features during the observation period.

First, in terms of location, the highest peak of the kernel density curve for 2010–2020 moves to the right, showing a significantly improved HSE. Second, in terms of the distribution profile, the height of the major peak of the distribution curve rises significantly, while the width narrows slightly, indicating a tendency for the absolute difference to expand. In other words, the level of HSE has gradually dispersed among the provinces, and the number of provinces that deviate from the average has gradually increased. Third, in terms of the extension of the curve, there is an obvious left trailing phenomenon in the kernel density curve, indicating that the difference in the efficiency of China’s healthcare services between high-level provincial areas (e.g., Beijing and Shanghai) and low-level provincial areas (e.g., Tibet and Ningxia) has expanded. Fourth, in terms of the polarization tendency, the peaks and waves of the curve maintain a “double-peak” state throughout the study period. Specifically, in 2010, the curve has a slight bimodal trend, indicating a polarization in the level of the HSE in China. By 2015, the bimodal distribution of efficiency has strengthened, with the efficiency level corresponding to the wave crest increasing compared with 2010. By 2020, the efficiency level corresponding to the wave crest has risen further, finally showing a robust polarization.

### Temporal evolution characteristics of China’s HSE

#### Traditional Markov chain analysis

Based only on the measured analysis of the HSE and temporal series static analysis, we cannot reflect the characteristics of the HSE shift over time and its probability. Therefore, we constructed the probability matrix of state transfer using the Markov chain model to study the changes in HSE transfer probability in different periods and categories. At the same time, by comparing the transfer probabilities, we can also discover the change pattern of HSE under different geospatial factors and clarify the influence of geospatial factors on different efficiency types of provinces. As shown in Table 8, the HSE levels of the 31 provinces in 2010–2020 were classified into four different categories based on quartiles. Specifically, the low levels (< 25%) are described as I; the medium–low levels (25–50%) as II; the medium–high levels (50–75%) as III; and the high levels (> 75%) as IV.

**Table 8:** Traditional Markov dynamic transformation probability matrix of the HSE in China from 2010 to 2020

t/t + 1	N	I	II	III	IV
		< 25%	25%–50%	50%–75%	> 75%
I	80	0.938	0.063	0.000	0.000
II	77	0.000	0.896	0.104	0.000
III	77	0.000	0.000	0.948	0.052
IV	76	0.000	0.000	0.000	1.000

I, II, III, and IV represent the four groups of the low level, medium–low level, medium–high level, and high level, respectively; N is the number of samples

The diagonal components in Table 8 indicate the probabilities of type invariance, revealing the stability of the HSE. Moreover, the shift probabilities between different types are represented by non-diagonal components. On this basis, the dynamic transfer trends of the HSE were obtained when the spatial spillover effects were not considered. First, the dynamic transfer probability is smaller on the non-diagonal elements than on the diagonal elements. To be specific, the probabilities of type I, II, III, and IV remaining intact are 93.8%, 89.6%, 94.8%, and 100%, respectively; the transfer probability of type I to type II is 6.3%; the transfer probability of type II to type III is 10.4%; and the transfer probability of type III to type IV is 5.2%. The findings indicated that the HSE in China remained stable over the study period. In other words, mobility between groups was minimal, particularly in the high-level areas. Second, the level of the HSE in China displays a clear upward trend. To be specific, the probability of a type II upward shift is 10.4%, and the probability of a type III upward shift is 5.2%. Both of these probabilities are higher than the probability of a downward shift. Third, transfers only occur in adjacent states, and it is difficult to make jump transfers (e.g., from a low-efficiency state to a high-efficiency state). This indicates that although China is actively pursuing healthcare reform policies, the probability of a leapfrog shift in the HSE is extremely low, and its enhancement is a long-term and relatively stable process.

#### Spatial Markov chain analysis

By comparing the traditional Markov dynamic transformation probability matrix with the spatial Markov dynamic transformation probability matrix, we could investigate the influence of the surrounding region on the probability of category conversion of a particular spatial unit’s HSE. The calculated results are shown in Table 9.

**Table 9:** Spatial Markov dynamic transformation probability matrix of the HSE in China from 2010 to 2020

Spatial Lag	t/t + 1	N	I	II	III	IV
			< 25%	25%–50%	50%–75%	> 75%
I	I	29	0.931	0.069	0.000	0.000
	II	16	0.000	1.000	0.000	0.000
	III	5	0.000	0.000	1.000	0.000
	IV	10	0.000	0.000	0.000	1.000
II	I	29	0.931	0.069	0.000	0.000
	II	25	0.000	0.920	0.080	0.000
	III	18	0.000	0.000	0.944	0.056
	IV	11	0.000	0.000	0.000	1.000
III	I	12	0.917	0.083	0.000	0.000
	II	28	0.000	0.857	0.143	0.000
	III	22	0.000	0.000	0.909	0.091
	IV	40	0.000	0.000	0.000	1.000
IV	I	10	1.000	0.000	0.000	0.000
	II	8	0.000	0.750	0.250	0.000
	III	32	0.000	0.000	0.969	0.031
	IV	15	0.000	0.000	0.000	1.000

I, II, III, and IV represent the four groups of low-level, medium–low level, medium–high level, and high- level, and N is the number of samples

As can be seen from the table, first, the transfer of the HSE in China is significantly affected by spatial factors. Specifically, the transfer probability of type I to type II is 6.3% when the spatial factor is not considered. Nevertheless, when spatial factors are considered, the probabilities of type I to type II are 6.9%, 6.9%, 8.3%, and 0%. Second, except for type IV, the stability of the HSE differs remarkably depending on the type of adjacency. Specifically, the transfer probability that type II is stable in adjacent types I, II, III, and IV is 100%, 92%, 85.7%, and 75%, respectively; the probability that type III is stable in adjacent types I, II, III, and IV is 100%, 94.4%, 90.9%, and 96.9%, respectively. The results reveal that the stability of HSE degrades with increasing spatial lag type by considering the spatial factors. Third, when a province is adjacent to a province with low HSE, the probability of the province’s HSE type decreasing increases; when a province is adjacent to a province with high HSE, the probability of the province’s HSE type rising increases. For example, the probability of upshifting is 10.4% on average for type II (Table 8), while it increases to 14.3% when adjacent to type III efficiency, and decreases to 8% when adjacent to low efficiency type. Thus, the transfer of HSE types in China is influenced by the spillover effects of neighboring types, and there is a “club convergence” phenomenon. In other words, the probability of an upward shift of a province can be increased when it is adjacent to a high-level province. Specifically, the probability of type II upward migration with increasing neighbor type is 0, 8%, 14.3%, and 25%. This indicates that when the HSE is higher in a neighboring region, there is a positive spatial spillover effect. This is because high-level areas have more developed economies, more complete healthcare facilities, and higher levels of medical technology and management, so the HSE in these provinces is bound to remain at a higher level, which in turn forms a stronger positive radiation drive to neighboring areas [111]. Therefore, the positive spillover from high-level regions should be valued.

### Spatial evolution characteristics of China’s HSE

#### Spatial correlation analysis

By analyzing the temporal evolution of the HSE in China, we can see that Chinese healthcare services show a bipolar distribution of high and low agglomerations and that spatial factors have a major influence on the transfer of efficiency. Thus, we used Stata software to calculate the global Moran’s I values of the HSE in 31 Chinese provinces from 2010 to 2020 to investigate the spatial correlation of the HSE and its spatial agglomeration characteristics in China. The results are shown in Table 10.

**Table 10:** Global autocorrelation Moran's I index of the HSE in China from 2010 to 2020

YEAR VALUE	Moran's I	*P*
2010–2012	0.042	0.016^**^
2013–2015	0.093	0.007^**^
2016–2018	0.109	0.045^**^
2019–2020	0.243	0.014^**^

^*^is significant at the 10% level, **is significant at the 5% level, ***is significant at the 1% level

As seen from the table, we can find that the global Moran’s I values for all years during the observation period are more significant than 0 and pass the significance test. This indicates that the spatial distribution of the HSE in China is positively correlated rather than distributed randomly. In addition, Moran’s I value climbs from 0.042 in 2010–2012 to 0.243 in 2019–2020, showing that spatial clustering increased yearly during the study period, while the positive spatial correlation of efficiency did not significantly decline.

#### Local autocorrelation analysis

**Fig. 4:**
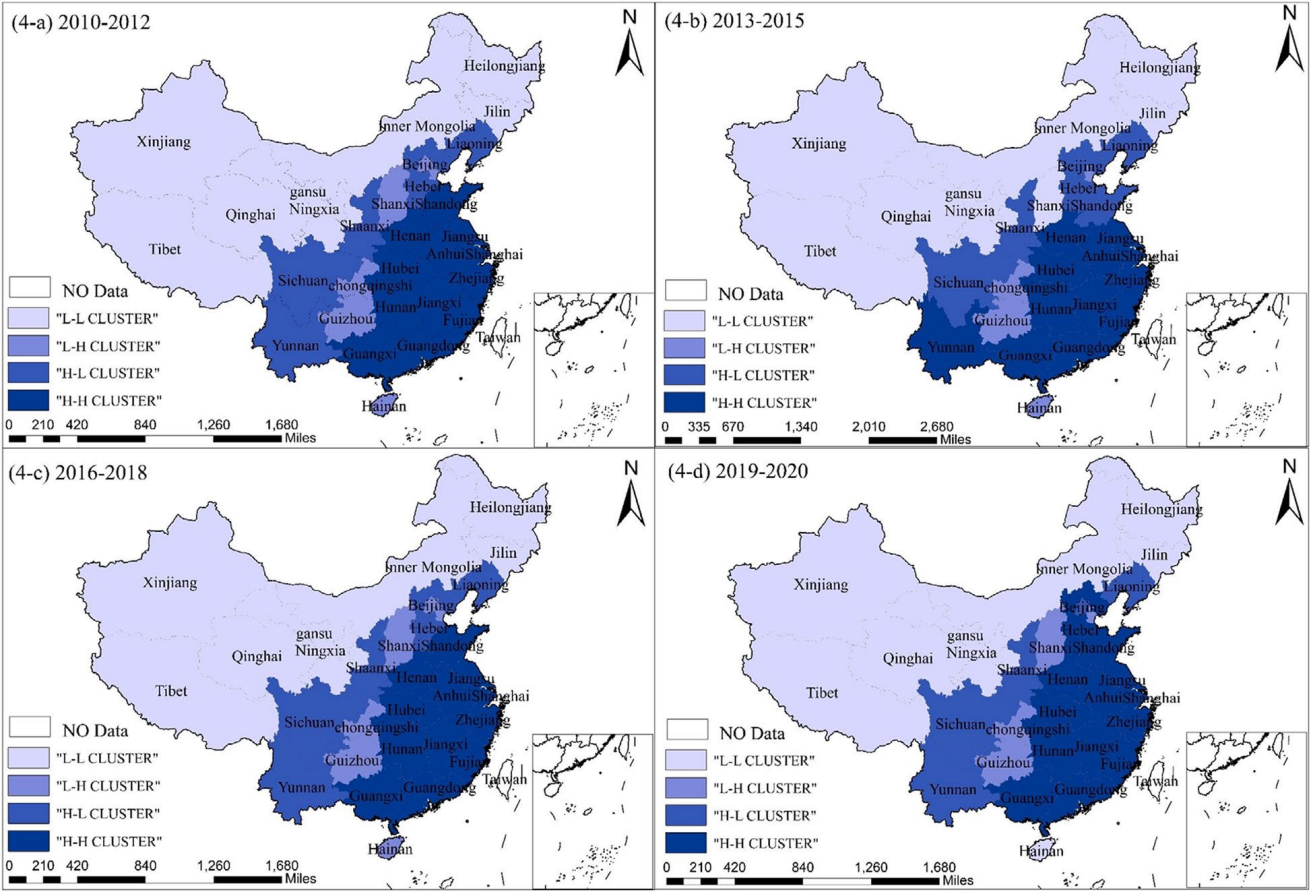
Spatial agglomeration characteristics of healthcare services efficiency in China from 2010 to 2020. **a** presents the spatial agglomeration characteristics of healthcare services efficiency in China from 2010 to 2012; **b** shows the spatial agglomeration characteristics of healthcare services efficiency in China from 2013 to 2015; **c** depicts the spatial agglomeration characteristics of healthcare services efficiency in China from 2016 to 2018; **d** exhibits the spatial agglomeration characteristics of healthcare services efficiency in China from 2019 to 2020

The global Moran’s I statistic can only confirm the level of the overall correlation of the HSE. Therefore, the years 2010–2012, 2013–2015, 2016–2018, and 2019–2020 were selected as the study’s research objects. Moreover, ArcGIS software was used to visualize each province’s efficiency clustering status in China. These include the H–H and L-L kinds, which point to a tendency of homogenous regional growth and exhibit some positive connections. By contrast, the H–L and L–H kinds show a negative correlation and reflect heterogeneous development tendencies among locations. The results are shown in Fig. 4.

Figure 4 demonstrates the spatial evolution of the HSE in China throughout the observation years. The agglomeration features display the presence of positive and negative spatial correlations. However, the regional homogeneity of efficiency development tendency becomes increasingly pronounced with time. The number of provinces in the region of positive spatial correlation increases from 20 provinces in 2010–2012 to 22 provinces in 2019–2020, accounting for 73.3%. This indicates that its positive spatial correlations are continuously increasing, which is consistent with the finding of the global autocorrelation test.

Specifically, the H–H type agglomeration is mainly concentrated in the eastern and central regions, including Jiangsu, Zhejiang, Hubei, Guangdong, and other provinces. This part of the region has its own efficient development of medical and health services, and at the same time, it drives the development of its neighboring provinces, showing a certain diffusion effect.

The L-L type agglomeration is mainly concentrated in the western region, which includes Tibet, Xinjiang, Inner Mongolia, Shanxi, and Jilin. Because this region is experiencing an efficiency “depression,” there is a substantial amount of negative spatial spillover, which primarily takes the form of the L-L type agglomeration. Yunnan, Sichuan, Shaanxi, and other provinces are included in the “strip-like” distribution of the H–L type agglomeration. Note that despite successfully developing its healthcare services, this area does not have a distinct diffusion effect. The L–H type agglomeration still exhibits a fragmented distribution, with Chongqing, Guizhou, Shanxi, Tianjin, and other provinces included. This also suggests that this area of the region is significantly less efficient than the areas around it. There are apparent “center-periphery” characteristics, which demonstrate a specific polarization effect. In addition, the tendency toward regional heterogeneity in efficiency development is diminishing. Specifically, the number of provinces with L–H and H–L agglomeration shows a downward trend, with only nine of such provinces by 2019–2020, accounting for 29.03%.

## Discussion

This was a comprehensive national study on the temporal-spatial evolution characteristics of the HSE in China. Unlike previous studies that focused on a certain local region [62, 111], we examined the variation and evolutionary characteristics of the HSE in the temporal-spatial dimensions across the country. In addition, unlike other research in the same category [43, 112, 113], we further investigated the dynamic transfer tendency, spatial spillover effects, and spatial agglomeration characteristics of the HSE in China using the kernel density estimation model, Markov chain model, and exploratory spatial data analysis. The associated research findings can contribute to the guidance of healthcare reform in the modern era. The principal findings are as follows.

First, regarding efficiency assessment, the annual average value of the HSE in China ranged from 0.83 to 0.851, and the overall level was not high, similar to the conclusions of most studies [114]. In terms of regions, the HSE in China had a decreasing distribution structure of “east-central-west,” with uneven development among regions. In fact, there is no consensus among scholars on the HSE of different regions. Most studies have confirmed that the eastern region has the highest HSE and that its health systems operate better than those in the central and western regions [115]. Nevertheless, a few scholars have argued that the HSE in the central and eastern regions is much lower than that in the western regions [105]. This may be closely related to the national support policies. To improve the development of health care and health status in the western region, the Chinese government has introduced preferential policies and invested significant resources [116]. Although existing research has surfaced different opinions on levels of HSE in different regions of China as a whole, they all concluded that there are apparent regional differences in HSE in China [8, 117], which agrees with the findings of this paper. The distinction in healthcare conditions is one explanation for this. Generally, the medical infrastructure in the eastern region is considered more developed and ideal, capable of meeting the population’s medical needs with greater efficiency and timeliness [118]. By contrast, the western part is hampered by a shortage of medical personnel and equipment and poor accessibility of medical resources and services [119]. Economic development is another reason. The ability of a region to finance improvements in public health care, technological infrastructure, and efficiency is related to its economic development [120]. However, the central and western regions have had slower economic growth. The lack of public funding has also limited the practical advancement of efficiency to some extent [121].

Moreover, the results of the MI analysis indicate that the TFP for healthcare services in China is increasing at a rate of 1.6% per year, which is similar to the findings of other studies [122]. A further breakdown of TFP change components suggests that the observed rise is primarily attributable to the rising techch. At the same time, the decline in effch has prevented an effective increase in TFP in Chinese healthcare services. Further decomposition also reveals that the decline in effch itself is caused by a decline in sech, which declines on average by about 1.2% per year. The decline in sech significantly offsets the improvement in pech observed during the period. Furthermore, the trends of techch and TFP were consistent, and it can be assumed that the changes in TFP were mainly affected by the changes in techch, which is consistent with the findings of Leng et al. [123]. Thus, it is reasonable to believe that technological advances determine the critical factors of TFP in Chinese healthcare services [124].

Second, in terms of temporal evolutionary characteristics, regarding temporal series static analysis, China’s HSE showed an upward trend, from 0.83 in 2010 to 0.851 in 2020. The existing literature has different views on the trends of the HSE in China. Several scholars have posited that HSE in China exhibits a slight downward trend [125]. However, the view of Wen et al. [43] is consistent with the findings of this study. We all agree that the HSE in China has been on an upward trend since 2010. The difference between high- and low-efficiency provinces of healthcare services in China has gradually expanded, forming a bipolar distribution of high and low agglomerations, with the extent of differentiation increasing yearly. This is entirely different from the findings of Li [61], which may be related to the different conclusions drawn by Li from the rural perspective. Regarding dynamic transfer trends, the probability of maintaining the original state of each province is greater than the transfer probability. Furthermore, China’s HSE has the stability to keep its state unchanged, making it difficult for efficiency changes to achieve leapfrogging, which is consistent with the findings of Chen [63]. After considering the spatial factor, we observed that the spatial transfer of efficiency states was active and that spatial spillover was prominent. Changes in efficiency states in neighboring regions had positive or negative spillover effects on the evolution of local efficiency states, which is similar to the findings of other studies [126]. Thus, localities need to pay attention not only to how to improve their HSE but also to the operating conditions and changing trends of the healthcare system in neighboring provinces to avoid the negative spatial impacts caused by them [127]. Positive spatial spillovers were observed in high-level provinces. When a province was neighbored by an area with high HSE, the likelihood of an upward shift of its HSE category was increased. One possible reason is that higher-efficiency provinces tend to perform better in terms of professional workforce, technological level, internal managerial experience, and socioeconomic development [128]. As inter-provincial economic exchanges become more frequent, the surplus capital, health technology, and sophisticated experience of efficient provinces often radiate to neighboring provinces. Thus, high-efficiency provinces had a significant positive spatial spillover effect, which is the same as the results of Wang [18]. This study examined the evolutionary characteristics of the HSE in China from a dynamic transfer probability perspective, which can help relevant government agencies monitor the HSE’s development dynamically.

Finally, in terms of spatial evolutionary characteristics, there was a significant positive spatial correlation of the HSE in China, the spatial agglomeration was dominated by homogeneous agglomeration (i.e., H–H or L-L agglomeration), and heterogeneous agglomeration (i.e., H–L or L–H agglomeration) tended to weaken. Specifically, the H–H agglomerations were mainly located in the eastern and central regions such as Shanghai, Zhejiang, Anhui, and Guangdong. The L-L agglomerations were mainly located in the western regions, such as Tibet and Qinghai. The H–L and L–H agglomerations were mainly located in the central and western regions, such as Yunnan, Shanxi, and Beijing. Over time, the spatial clustering of the HSE in China stabilized. Other scholars have conducted similar studies [62, 129]. Workforce mobility and social welfare coverage, such as Medicare programs, contribute to this geographical aggregation feature. In recent years, the demand for healthcare services in the eastern and central regions has significantly increased owing to people migrating from the west to the east in quest of wealth and opportunity [130]. However, in China, most provinces offer health insurance sharing, and insured people do not receive the same share of the money reimbursed from other types of risk sharing [131]. To acquire good health insurance coverage, many migrant workers choose to receive their medical treatment, including outpatient and inpatient services, in the eastern and central areas where they work [132, 133].

## Conclusions and policy recommendations

In recent decades, especially after the implementation of the “New Health Care Reform” in 2009, China has achieved significant progress in the field of healthcare services. Nevertheless, the healthcare system in some areas has continued to operate inefficiently, the mismatch between high health inputs and low health outputs has become increasingly prominent. In this context, it is of positive significance to accurately assess HSE in China, grasp the temporal-spatial dynamic evolution characteristics of the HSE, and provide valuable information to help policymakers improve the HSE and meet the growing demand for health care. Accordingly, we used a comprehensive SFA-Malmquist model to measure the overall level and dynamic sources of change in the HSE based on panel data from 31 Chinese provinces from 2010 to 2020. Furthermore, the kernel density estimation model, Markov chain model, and exploratory spatial data analysis were utilized to reveal the HSE’s temporal-spatial evolutionary features in China.

Our findings indicate that (1) China’s HSE is generally at a moderate level. The HSE of each region has apparent differences, presenting a decreasing characteristic of “East > Central > West.” (2) From 2010 to 2020, the TFP for healthcare services in China grew by 1.6% per year. This growth was driven by a 1.8% annual increase in technological progress (techch). (3) The level of the HSE in China has improved, and the difference between high-efficiency areas and low-efficiency areas has gradually expanded, forming a bipolar distribution of high and low agglomerations; China’s HSE has the stability to maintain its own state unchanged, and the probability of leapfrogging is low. (4) The temporal evolution of the HSE in China exhibits a clear spatial spillover effect, with high-efficiency provinces showing a marked positive spillover effect on neighboring provinces and low-efficiency provinces experiencing a negative spillover effect. Thus, the “club convergence” phenomenon of “high efficiency concentration, low efficiency agglomeration, high levels of radiation, and low levels of suppression” has formed. (5) From 2010 to 2020, the spatial distribution of the HSE in China had a positive spatial correlation, and the spatial agglomeration was dominated by homogeneous agglomeration (i.e., H–H or L-L agglomeration), while heterogeneous agglomeration (i.e., H–L or L–H agglomeration) tended to weaken.

According to the aforementioned research results, this paper puts forward the following strategies and recommendations to improve the HSE in China:

First, we should promote the Health China strategy and pay more attention to improving resource utilization efficiency based on ensuring the growth of the total supply of medical and health resources. In addition, the government should coordinate the regional allocation of high-quality medical and health resources following the level of regional economic development and the demand for medical services to address the current imbalance in regional development. The government should provide some financial, technical, and human resource support to the central and western regions and appropriately step up their policy support for the western regions.

Second, to improve the total factor productivity of China’s healthcare services, we should introduce high-tech medical equipment, improve the ability of medical and health technology innovation, strengthen the training mechanism of medical personnel and innovation capacity, and emphasize the importance of optimizing the “soft power” of healthcare institutions.

Third, the government should make flexible use of inter-provincial “spillover effects” to reduce differences in the HSE across provinces. High-efficiency level provinces should take the initiative to break the inter-provincial interest barriers and policy barriers to give full play to a radiation-driven role. Low-efficiency provinces should learn from neighboring provinces with higher-efficiency healthcare management experience and combine their development conditions to seek a balanced point of coordinated development between economic growth, health resource allocation, and HSE improvement, thus improving the efficiency gap of healthcare services with high-efficiency provinces.

Fourth, we must further implement a coordinated development strategy for regional public health. Specifically, the H–H agglomeration areas should make the most of their healthcare advantages and implement the regional assistance model. To do so, they should take the initiative to share the excellent system management practices and technical lessons learned with low-efficiency provinces; gradually realize the cross-regional sharing of highly qualified healthcare personnel, technology, knowledge, and other resources; and maximize the efficiency of healthcare services in the neighborhood. By contrast, L-L agglomeration areas can improve their healthcare systems in terms of organizational management and information technology by adopting successful strategies that can be applied to other regions. Then, they can maintain an optimal level of healthcare personnel and technical capability and encourage the ongoing enhancement of healthcare service efficiency by cooperative regional adjustments.

There were still some limitations to our research. First, the input and output variables in this study were chosen based on the relevant literature and data accessibility, potentially leading to bias in the research results. Second, the study and empirical analysis were carried out from a macro and overall standpoint, without a detailed study of each province’s characteristics and detailed descriptions of specific provinces. Finally, this study focused on analyzing China’s HSE and its temporal-spatial evolution patterns, and did not conduct a specific analysis of the factors that influence HSE. Thus, the selection of indicators, influencing factors, and characteristics of individual provinces will be examined in depth in the future.

## Data Availability

''The datasets generated and/or analysed during the current study are not publicly available due [them containing information that could compromise research participant and public privacy/consent] but are available from the corresponding author on reasonable request.''.

## Abbreviations

DEA: Data Envelopment Analysis
SFA: Stochastic frontier analysis
DMUs: Decision making units
MI: Malmquist Index
TFP: Total Factor Productivity
tfpch: Total Factor Productivity Change
effch: Technical Comprehensive Efficiency Change index
techch: Technical Change index
pech: Pure Efficiency Change index
sech: Scale Efficiency Change index
